# Supplementary material for: Insect Feeding on Sorghum bicolor Pollen and Hymenoptera Attraction to Aphid-Produced Honeydew
Source: Insects. 2022 Dec 14;13(12):1152. doi: 10.3390/insects13121152 (PMC9780982; doi:10.3390/insects13121152)
Supplement: Supplementary file 1 [file insects-13-01152-s001.zip › insects-2080408-supplementary.pdf]

Supplemental Data.

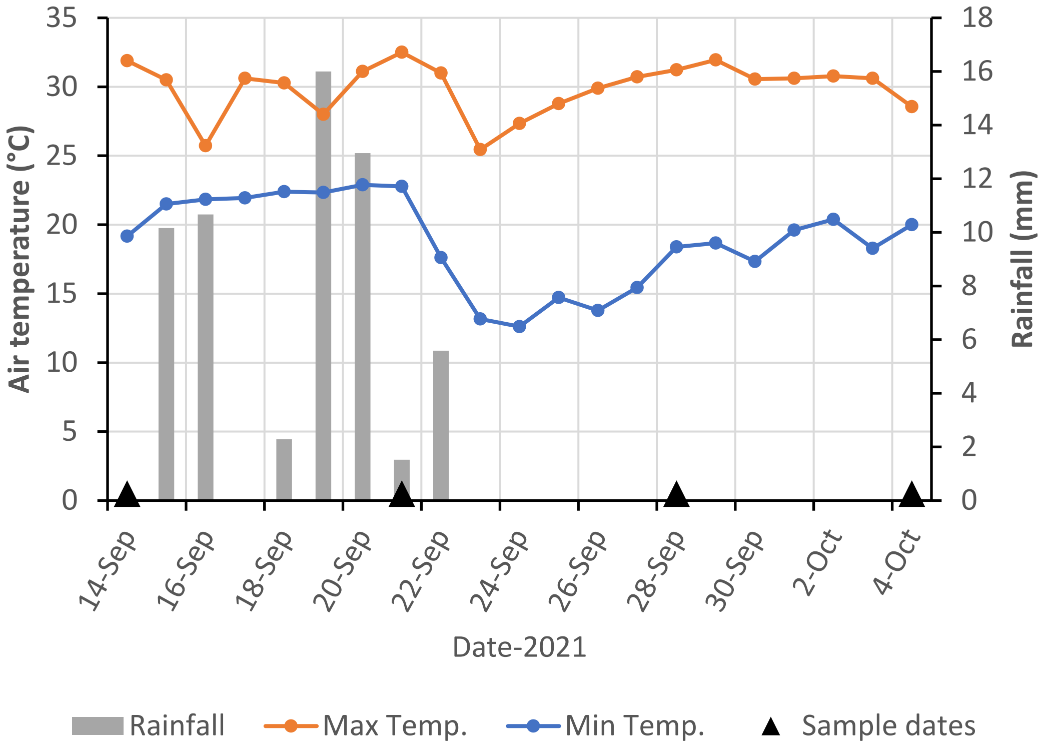

Supplemental Figure S1. Daily rainfall and maximum and minimum air temperatures during the study period in Tifton, GA in 2021. Data from the University of Georgia Weather Network (weather.uga.edu), Tifton station. Insect sampling dates are indicated by black triangles.

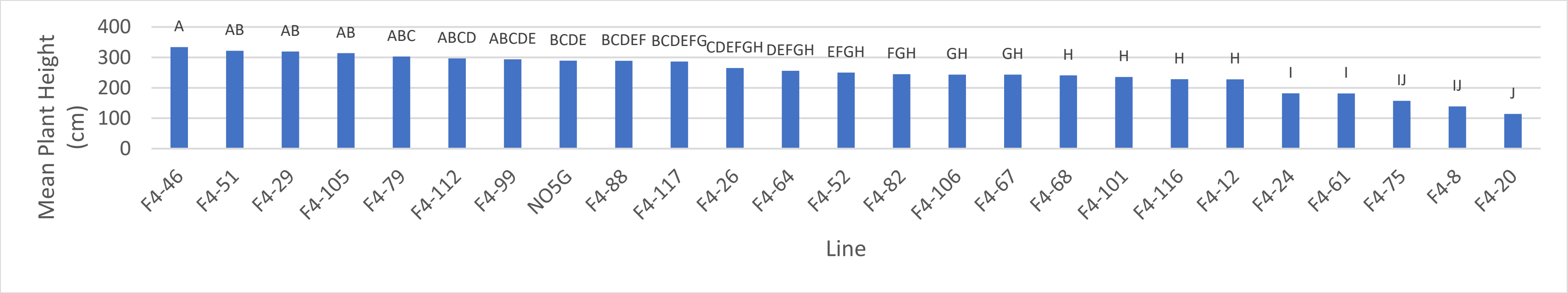

Supplemental Figure S2. Plant height for the N109A x PI 257599 F_4 sorghum lines grown at Tifton, GA 2021. NO5G is PI 257599 (the common name is No. 5 Gambela). Means with the same letter are not different at $\alpha = 0.05$.

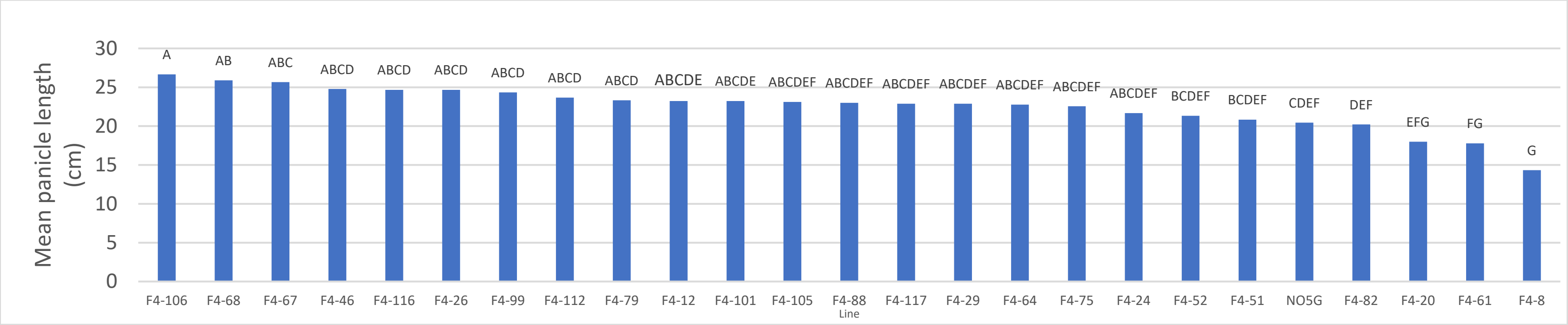

Supplemental Figure S3. Mean panicle length for the N109A x PI 257599 F_4 sorghum lines grown at Tifton, GA 2021. NO5G is PI 257599 (the common name is No. 5 Gambela). Means with the same letter are not different at $\alpha = 0.05$.
